# Supplementary material for: Industrial technology progress, digital finance development and corporate risk-taking: Evidence from China’s listed firms
Source: PLoS One. 2024 Mar 15;19(3):e0298734. doi: 10.1371/journal.pone.0298734 (PMC10942030; doi:10.1371/journal.pone.0298734)
Supplement: Attachment — Submitted filename: review comments.pdf [file pone.0298734.s002.pdf]

Dear author,

Based on the data of China's A-share listed companies from 2011 to 2020, the paper empirically investigates the impact of industrial technological progress on firms' risk-taking and the role played by digital financial development in it. The study finds that industrial technological progress promotes the increase in the level of corporate risk-taking. Digital financial development has a positive moderating effect on the relationship between industrial technological progress and the level of corporate risk-taking. Under the role of digital financial development, industrial technological progress has produced financing constraint alleviation effect, input capital return enhancement effect and innovation performance incentive effect. In addition, industrial technological progress has a significant impact on the level of corporate risk-taking in the eastern region and among non-high-tech firms, while digital financial development has a more pronounced positive moderating effect on the relationship between industrial technological progress and the level of risk-taking among firms in the central and western regions as well as among firms in high-tech industries. Throughout the existing research, the literature on the impact of industrial technological progress on firms' micro-behavior is extremely rare; therefore, the paper enriches the research literature on the microeconomic consequences of industrial technological progress and the factors influencing firms' risk-taking, and it also provides marginal evidence for the research literature on digital finance.

In conclusion, the theoretical study of this article on industrial technology is a very novel and interesting topic with research value and some innovative significance. But the article has some worthwhile improvements, as follows:

1. The split-sample test does not provide an analysis relevant to the topic, but only a split-sample

regression of the data, and the rationale for the split-sample needs to be further elaborated.

2. The article needs to make some additional explanations on the model construction.

3. The robustness test is too simple, it is recommended to add more tests to ensure the reliability of the conclusion.

4. The purpose is not clearly stated in the abstract, and the methodology is not described.

5. The analysis of the regression results is rather simple and does not analyze the deeper reasons behind the results.

6. the period 2011-2020 is the beginning of the covid pandemic, will the data be affected by this?

7. The spacing between words is inconsistent in some parts of the article, and the formatting needs to be improved.

Sincerely.
