# Supplementary material for: Industrial technology progress, digital finance development and corporate risk-taking: Evidence from China’s listed firms
Source: PLoS One. 2024 Mar 15;19(3):e0298734. doi: 10.1371/journal.pone.0298734 (PMC10942030; doi:10.1371/journal.pone.0298734)
Supplement: Attachment — Submitted filename: Response to Reviewer.doc [file pone.0298734.s003.doc]

**Author Responses to Reviewer #1’s Comments on Manuscript # PONE-D-23-41757,**

**“Industrial Technology Progress ,Digital Finance Development**

**And Corporate Risk-Taking: Evidence from China's listed firms”**

We appreciate your time and effort in carefully reading our paper and providing helpful feedback. We have endeavored to think carefully about each of the points you raise and hope you will agree that the paper has made substantial progress. We reproduce the comments and suggestions from your report below and respond to each in turn.

1.*There is no literature reference for measuring industrial technological progress by total factor productivity, so it is suggested to supplement.*

Our Response:Thanks to your suggestions, this article has been revised and the changes are marked in red.

2.*Benchmarking model, moderating effect model, etc. only have equations without more detailed explanations of variables.*

Our Response:Thanks to your suggestions, this article has been revised and the changes are marked in red.

3.*The research sample is fine, but there is no literature background in section 3.1.*

Our Response:Thanks to your suggestions, this article has been revised and the changes are marked in red.

4.*The comprehensibility of the tables in the paper is low, mainly because of the following problems: (1) none of the tables indicate the dependent variable; (2) the tables are disclosed without textual explanations.*

Our Response:Thanks to your suggestions, this article has been revised and the changes are marked in red.

5. *It is suggested that the endogeneity test should be further supplemented.*

Our Response:The paper has been tested for endogeneity using the instrumental variables approach and the results show that the article's conclusions are reliable and robust

6.*Although the case of China is presented, there is no explanation as to whether this is a global problem for many countries, which warrants a study of the Chinese case as its justification.*

Our Response:Thank you for your suggestions. Some of the theories specific to China have been modified and deleted from this article.

7. *Are there any limitations to the thesis? What about future research perspectives?*

Our Response:Thanks to your suggestions, this article has been revised and the changes are marked in red.

8. *Some statements in the essay need to be improved and standardize the terminology.*

Our Response:Thank you so much for your careful check.this article has been modified.

**Author Responses to Reviewer #2’s Comments on Manuscript # PONE-D-23-41757,**

**“Industrial Technology Progress ,Digital Finance Development**

**And Corporate Risk-Taking: Evidence from China's listed firms”**

1. *The split-sample test does not provide an analysis relevant to the topic, but only a split-sample regression of the data, and the rationale for the split-sample needs to be further elaborated.*

Our Response:Thanks to your suggestions, this article has been revised and the changes are marked in red.

2. *The article needs to make some additional explanations on the model construction.*

Our Response:Thanks to your suggestions, this article has been revised and the changes are marked in red.

3. *The robustness test is too simple, it is recommended to add more tests to ensure the reliability of the conclusion.*

Our Response:Thank you for your suggestion. In this paper, we intercepted a subsample for robustness tests to exclude the effect of  *covid pandemic* on the data.

4. *The purpose is not clearly stated in the abstract, and the methodology is not described.*

Our Response:Thank you for your suggestions, this article has been modified

5. *The analysis of the regression results is rather simple and does not analyze the deeper reasons behind the results.*

Our Response:Thanks to your suggestions, this article has been revised and the changes are marked in red.

6. *The period 2011-2020 is the beginning of the covid pandemic, will the data be affected by this?*

Our Response:In this paper, the regression was resumed by excluding the years in which the epidemic occurred, and the results are consistent with the previous paper, thus proving that the results of this paper are reliable.

7. *The spacing between words is inconsistent in some parts of the article, and the formatting needs to be improved.*

Our Response:Thank you so much for your careful check.this article has been modified.
